# Metabolite Profile Resulting from the Activation/Inactivation of 1-Methyl-4-phenyl-1,2,3,6-tetrahydropyridine and 2-Methyltetrahydro-****β****-carboline by Oxidative Enzymes

**DOI:** 10.1155/2013/248608

**Published:** 2013-07-28

**Authors:** Tomás Herraiz, Hugo Guillén, Juan Galisteo

**Affiliations:** Instituto de Ciencia y Tecnología de Alimentos y Nutrición (ICTAN), Consejo Superior de Investigaciones Científicas (CSIC), Juan de la Cierva 3, 28006 Madrid, Spain

## Abstract

Metabolic enzymes are involved in the activation/deactivation of the 1-methyl-4-phenyl-1,2,3,6-tetrahydropyiridine (MPTP) neurotoxin and its naturally occurring analogs 2-methyltetrahydro-**β**-carbolines. The metabolic profile and biotransformation of these protoxins by three enzymes, monoamine oxidase (MAO), cytochrome P450, and heme peroxidases (myeloperoxidase and lactoperoxidase), were investigated and compared. The metabolite profile differed among the enzymes investigated. MAO and heme peroxidases activated these substances to toxic pyridinium and **β**-carbolinium species. MAO catalyzed the oxidation of MPTP to 1-methyl-4-phenyl-2,3-dihydropyridinium cation (MPDP^+^), whereas heme peroxidases catalyzed the oxidation of MPDP^+^ to 1-methyl-4-phenylpyridinium (MPP^+^) and of 2-methyltetrahydro-**β**-carboline to 2-methyl-3,4-dihydro-**β**-carbolinium cation (2-Me-3,4-DH**β**C^+^). These substances were inactivated by cytochrome P450 2D6 through *N*-demethylation and aromatic hydroxylation (MPTP) and aromatic hydroxylation (2-methyltetrahydro-**β**-carboline). In conclusion, the toxicological effects of these protoxins might result from a balance between the rate of their activation to toxic products (i.e., *N*-methylpyridinium-MPP^+^ and MPDP^+^- and *N*-methyl-**β**-carbolinium—**β**C^+^—) by MAO and heme peroxidases and the rate of inactivation (i.e., *N*-demethylation, aromatic hydroxylation) by cytochrome P450 2D6.

## 1. Introduction

**Figure 1:**
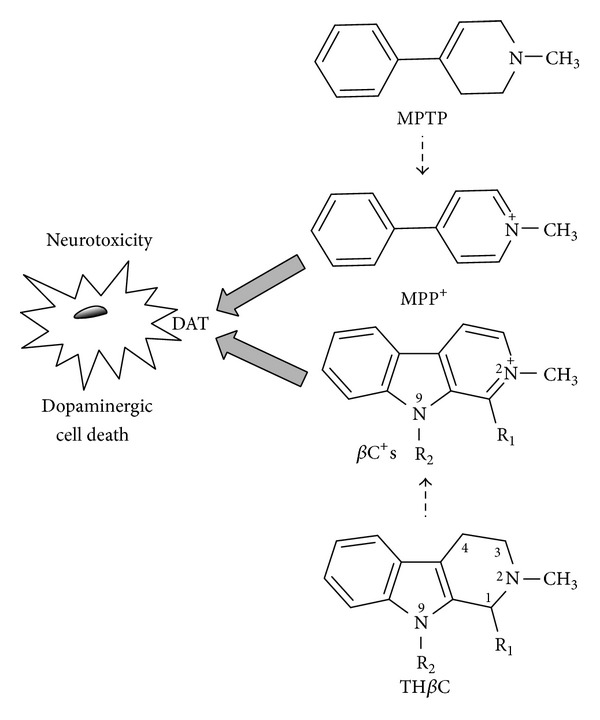
Activation of MPTP to the directly acting neurotoxin MPP^+^, which is uptaken by dopaminergic cells via DAT (dopamine active transporter) and produces neurotoxicity and cell death. *β*-Carbolinium cations (*β*C^+^) are toxic analogs of MPP^+^ that may arise from 2-methyl-1,2,3,4-tetrahydro-*β*-carbolines.

The causative factors of neurodegenerative diseases such as Parkinson's disease (PD) remain unknown, although the involvement of environmental and/or endogenous neurotoxins is being increasingly considered [1–3]. Exposure to 1-methyl-4-phenyl-1,2,3,6-tetrahydropyridine (MPTP), a contaminant found in “synthetic heroin,” produces neurotoxicity in humans, and this neurotoxin is commonly used to generate experimental parkinsonism in animal models [4–7]. MPTP crosses the blood-brain barrier and is bioactivated enzymatically to give 1-methyl-4-phenylpyridinium (MPP^+^) [1, 8, 9], which is selectively uptaken into dopaminergic cells via dopamine-activated transporter (DAT) and produces inhibition of mitochondrial complex I, energy depletion, and cell death [8] (Figure 1). Besides its use in experimental models of neurotoxicity, the toxic outcome caused by MPTP is a matter of investigation due to the differences in response among experimental models [10, 11]. This might result from a change in the balance between the rate of metabolism to toxic products (MPP^+^ and MPDP^+^) (activation) and the rate of detoxification (inactivation) [12–16]. MPTP is metabolized by enzymes such as cytochrome P450, and this could affect the outcome of this neurotoxin [12, 14, 17–19]. 

Humans are not usually exposed to MPTP but are exposed to some structural analogs such as the *β*-carboline alkaloids [20–22]. *β*-Carbolines appear in foods and are absorbed into the human body and brain, where they may exert psychopharmacological and toxic effects [23–28]. These compounds might follow a toxicological pattern similar to that of MPTP (Figure 1). Firstly, these alkaloids could be bioactivated to *N*-methyl derivatives and then oxidized (aromatized) to pyridinium-like *β*-carbolinium species [15, 20, 21, 29]. *β*-Carbolinium (*β*C^+^) species share several functional and toxicological properties with 1-methyl-4-phenylpyridinium (MPP^+^), which is the metabolite involved in MPTP neurotoxicity [30–32], and were postulated as potential slow-acting neurotoxins [33, 34]. Remarkably, *N*-methyl-*β*-carbolinium species such as *N*-methylnorharmanium (2-Me-*β*C^+^) and 2,9-dimethylnorharmanium (2,9-diMe-*β*C^+^) have been detected in postmortem human brains [34–36] and found in higher proportion in cerebrospinal fluid of patients with neurodegenerative diseases (PD) [35]. 

The toxicological outcome of MPTP and *β*-carbolines will depend on the metabolic profile produced by key enzymes leading to the activation/inactivation of these protoxins [21]. Therefore, studying the activation/inactivation (biotransformation) of MPTP and its naturally occurring analogs by metabolic enzymes is a matter of current interest in order to explain the toxicological features of these substances. It could lead to the identification of the enzyme responsible for activation and inactivation as well as the metabolites produced, and it may also suggest interindividual differences. In this regard, the purpose of this research was to study in a comparative way the metabolic profile generated from MPTP and its naturally occurring analog 2-methyltetrahydro-*β*-carboline by three metabolic enzymes: monoamine oxidase (MAO), heme peroxidase, and cytochrome P450 (2D6). Monoamine oxidase (MAO) is a flavoenzyme located at the outer membranes of mitochondria in the human brain and peripheral tissues that catalyzes the oxidative deamination of neurotransmitters and xenobiotic amines. MAO appears as two isozymes, MAO-A and B, and plays an important role in the central nervous system and peripheral organs [37]. MAO-A is involved in psychiatric conditions and depression and MAO-B is implicated in neurodegenerative diseases [37–41]. The cytochrome P450 enzymes are mixed-function oxidases involved in the metabolism of drugs and xenobiotics. In particular, the cytochrome P450 2D6 is present in the liver and extrahepatic tissues and participates in the metabolism and toxicity of many drugs with a basic nitrogen. This cytochrome presents strong polymorphism, characterized by poor, intermediate, extensive, and ultrarapid metabolizers, and it is currently being considered in relation to neurodegenerative diseases [17, 42–46]. Heme peroxidases participate in the oxidation of endogenous substrates, drugs, and xenobiotics [47]. Mammalian peroxidases such as myeloperoxidase (MPO), eosinophil peroxidase (EPO), and lactoperoxidase (LPO) are found in neutrophils, eosinophils, and secretory cells of the exocrine glands and participate in antimicrobial and anti-inflammatory processes. MPO occurs in activated microglia at sites of degenerative diseases [48–50]. Peroxidases in the substantia nigra may produce toxic substances and might be involved in PD and neurodegeneration [51, 52]. 

## 2. Material and Methods

### 2.1. Chemicals and Enzymes

1-Methyl-4-phenyl-1,2,3,6-tetrahydropyridine (MPTP) hydrochloride (caution: MPTP is a neurotoxin and should be handled with appropriate precautions), 1-methyl-4-phenyl-2,3-dihydropyridinium (MPDP^+^) perchlorate, 1-methyl-4-phenylpyridinium (MPP^+^) iodide, 4-phenyl-1,2,3,6-tetrahydropyridine hydrochloride (PTP), and NADPH were from Sigma-Aldrich (St. Louis, MO, USA). 2-Methyl-1,2,3,4-tetrahydro-*β*-carboline hydrochloride (2-Me-TH*β*C), 2-methyl-3,4-dihydro-*β*-carbolinium chloride (2-Me-3,4-DH*β*C^+^), 2-methyl-*β*-carbolinium iodide (2-Me-*β*C^+^), and 4-(4′-hydroxyphenyl)-1-methyl-1,2,3,6-tetrahydropyridine hydrochloride (MPTP-OH) were obtained previously [14–16]. Human monoamine oxidases (MAO-A and -B) were obtained from BD Gentest Co. (Woburn, MA, USA). Microsomes containing recombinant human cytochrome P450 2D6*1+cytochrome P450 oxidoreductase produced from baculovirus infected-insect cells were obtained from BD Gentest Co. (Woburn, MA, USA). Bovine lactoperoxidase (LPO) and human myeloperoxidase (MPO) were obtained from Sigma and Calbiochem (Merck), respectively, and the concentration in the assays is determined using the extinction coefficients of Soret bands [53]. 

### 2.2. Enzyme Biotransformation and Metabolic Profile

#### 2.2.1. MAO Enzymes

 0.2 mL reaction mixtures in 75 mM buffer phosphate (pH 7.4) containing human MAO-A or -B (0.01–0.2 mg/mL protein) and MPTP (50, 250 *μ*M) or 2-methyl-1,2,3,4-tetrahydro-*β*-carboline (50, 250 *μ*M) were incubated (37°C, 40 min); the reaction was stopped by the addition of 2 N NaOH (75 *μ*L) and 70% perchloric acid (25 *μ*L) subsequently centrifuged (9000 rpm, 5°C), and 20 *μ*L of the supernatant injected into the HPLC. The metabolic profile was analyzed by HPLC-DAD, and the metabolites identified by mass spectrometry (ESI). Incubations were performed at least in duplicate. 

#### 2.2.2. Cytochrome P450 2D6

 0.2 mL reaction mixtures in 75 mM phosphate buffer (pH 7.4) containing human cytochrome P450 2D6 (7 pmol P450) and MPTP (50, 250 *μ*M) or 2-methyl-1,2,3,4-tetrahydro-*β*-carboline (50, 250 *μ*M), and NADPH (1 mM) were incubated at 37°C, 25 min. The reaction was stopped with a mixture of methanol and perchloric acid (1 : 1) (50 *μ*L), centrifuged at 10000 rpm for 10 min, and 5°C, and 20 *μ*L of the supernatant injected into the HPLC. The metabolic profile was analyzed by HPLC-DAD, and the metabolites identified by mass spectrometry (ESI). Incubations were performed at least in duplicate.

#### 2.2.3. Heme Peroxidases

 0.5 mL reaction mixtures in 50 mM phosphate buffer (pH 7), containing lactoperoxidase (LPO) (0.18 *μ*M) or myeloperoxidase (MPO) (0.013 *μ*M), and MPTP (50, 250 *μ*M), MPDP^+^ (50, 250 *μ*M) or 2-methyl-1,2,3,4-tetrahydro-*β*-carboline (50, 250 *μ*M), and H_2_O_2_ (25 *μ*M) were incubated at 37°C, 40 min. Following the addition of HClO_4_ + methanol (1/1) (10% v/v of reaction volume), the tubes were centrifuged at 10000 rpm, 10 min, and 20 *μ*L of the supernatant injected into the HPLC. The metabolic profile was analyzed by HPLC-DAD, and the metabolites identified by mass spectrometry (ESI). Incubations were performed at least in duplicate.

#### 2.2.4. RP-HPLC Chromatographic Analysis and Mass Spectrometry

 The chromatographic analysis of the reaction products from enzyme incubations was performed by RP-HPLC with *uv-DAD *and fluorescence detection using an HPLC 1050 (Hewlett Packard) with a Diode Array Detector (DAD) and a 1046A-fluorescence detector [14, 15]. A 150 mm × 3.9 mm, 4 *μ*m, Nova-pak C18 column (Waters, Milford, MA, USA) was used for chromatographic separation. Chromatographic conditions were buffer A: 50 mM ammonium phosphate buffer (pH 3 for MAO and peroxidase assays or pH 5.5 for cytochrome P450 2D6) and buffer B: 20% of A in acetonitrile. Gradient was programmed from 0% (100% A) to 32% B at 8 min and 90% B at 15 min. The flow rate was 1 mL/min, the column temperature was 40°C, and the injection volume was 20 *μ*L. Absorbance detection was set at 355 nm for analysis of dehydrogenation products such as MPDP^+^ and 2-methyl-3,4-dihydro-*β*-carbolinium species (2-Me-DH*β*C^+^); 280 nm for analysis of MPP^+^; 254 nm for the analysis of 2-methyl-*β*-carbolinium cation; 280 nm for 2-methyltetrahydro-*β*-carboline (2-Me-TH*β*C) and its metabolites, 243 nm for PTP, and 254 nm for MPTP-OH. Calibration curves of absorbance versus concentration were constructed for each metabolite. Identification of metabolites was done by UV (DAD spectra) fluorescence and coelution with authentic standards. Confirmation of the identity was performed with HPLC-ESI-mass spectrometry [14, 15]. For that, separation was accomplished on a 2.1 × 150 mm Zorbax SB-C18 3.5 *μ*m column by using an HPLC-MSD Series 1100 (Hewlett Packard, Santa Clara, CA, USA) working under electrospray ionization positive-ion mode. Eluents: (A) formic acid (0.5%), (B) formic acid 0.5% in acetonitrile; 80% B in 30 min, flow: 0.25 mL/min, cone voltage: 70 V, and mass range: 50–600 amu.

## 3. Results and Discussion

**Figure 2:**
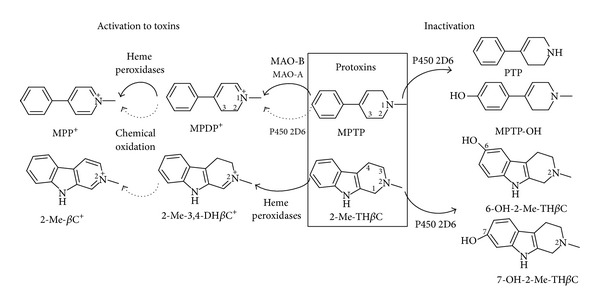
Proposed activation and inactivation routes and metabolites from MPTP and 2-methyl-1,2,3,4-tetrahydro-*β*-carboline (2-Me-TH*β*C) protoxins resulting from human monoamine oxidase (MAO), heme peroxidases (lactoperoxidase and myeloperoxidase), and human cytochrome P450 2D6.

**Figure 3:**
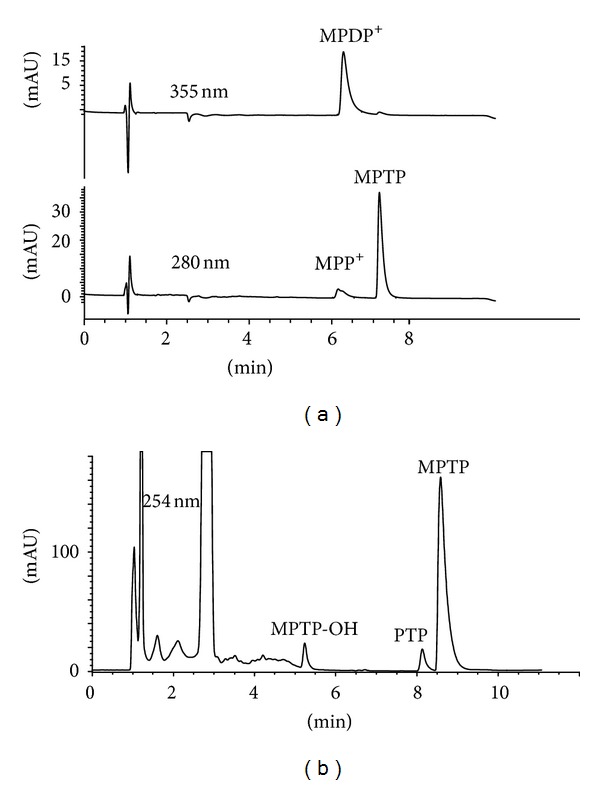
HPLC chromatograms of MPTP oxidized by human MAO-B (a) and MPTP oxidized by human cytochrome P450 2D6 (b). Enzyme assays and chromatographic conditions were as indicated in experimental section.

**Figure 4:**
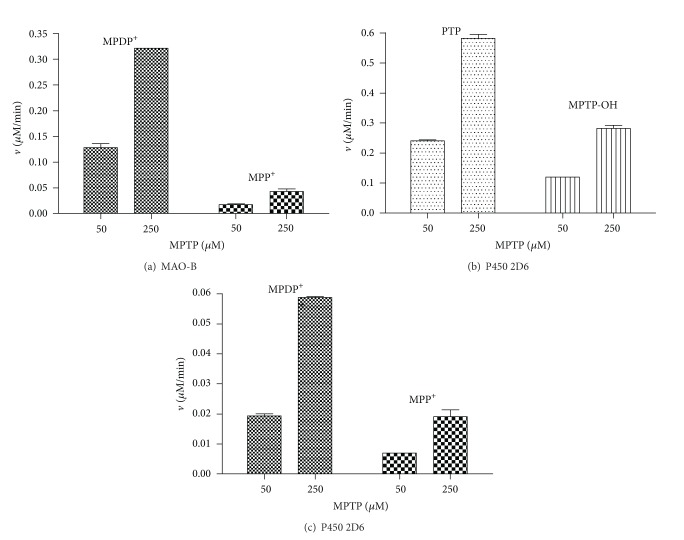
Metabolites and rates produced from MPTP neurotoxin (50 and 250 *μ*M) by human MAO-B (0.05 mg/mL protein) (a) and human cytochrome P450 2D6 (b) and (c). Enzyme assays were as indicated in experimental section.

The activation and inactivation of MPTP neurotoxin and 2-methyltetrahydro-*β*-carboline protoxin occur with the participation of key metabolic enzymes. This research studied and compared the metabolic profile generated from these substances by human monoamine oxidase, human cytochrome P450 2D6, and heme peroxidases (Figure 2). Human MAO enzymes (MAO-B) oxidized MPTP to give MPDP^+^ and MPP^+^ (Figure 3(a)). The main metabolite arising from MAO and MPTP was MPDP^+^, whereas MPP^+^ was produced through subsequent oxidation of MPDP^+^ (Figure 4(a)). As the pyridinium species are the directly acting neurotoxins in vivo, the oxidation by MAO is considered a key route for the bioactivation of MPTP (Figure 2) [8]. Indeed, inhibitors of MAO-B usually protect against this neurotoxin and can be useful as neuroprotectants [37, 40, 41, 54, 55]. Although human MAO-A was also able to oxidize MPTP in vitro as well, a number of studies have shown that MAO-B is the main isoform involved in this oxidation [54–57].

The neurotoxin MPTP was metabolized by the cytochrome P450 2D6 (Figure 3(b)). Two major metabolites were 4-(4′-hydroxyphenyl)-1-methyl-1,2,3,6-tetrahydropyridine (MPTP-OH) (aromatic hydroxylation) and 4-phenyl-1,2,3,6-tetrahydropyridine (PTP) (*N*-demethylation) (Figure 4(b)). In addition, the cytochrome P450 2D6 oxidized MPTP to give minor amounts of MPDP^+^ and MPP^+^, although this conversion was of lower efficiency compared with MAO (Figure 4(c)). On the other hand, two heme peroxidases (lactoperoxidase and myeloperoxidase) were unable to oxidize MPTP to pyridinium species in the presence of H_2_O_2_, indicating that MPTP was not a substrate of these kinds of oxidative enzymes. Interestingly, however, these peroxidases accelerated the oxidation of MPDP^+^ to MPP^+^ (Figure 6(c)). Although MPDP^+^ could be auto oxidized or disproportionated to give MPP^+^ [15, 58] as reported in Figure 2, peroxidases increased this oxidation when compared to controls. Then, activation of the MPDP^+^ to the directly acting neurotoxin MPP^+^ could be facilitated by heme peroxidases, and this might have further implications for the neurotoxicity of this and related substances (Figure 2).

**Figure 5:**
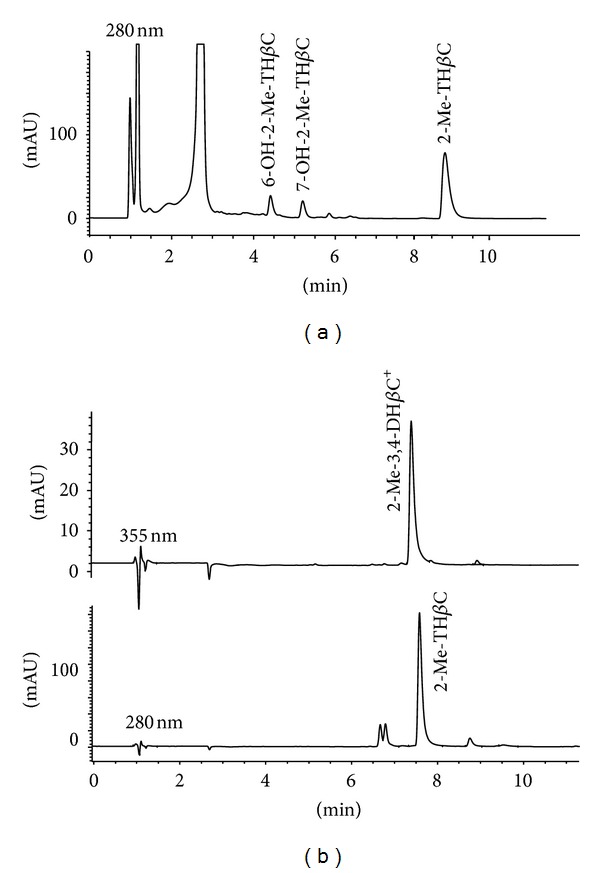
HPLC chromatograms of metabolites from 2-methyl-1,2,3,4-tetrahydro-*β*-carboline incubated with cytochrome P450 2D6 (a) and myeloperoxidase (b). Enzyme assays and chromatographic conditions were as indicated in experimental section.

**Figure 6:**
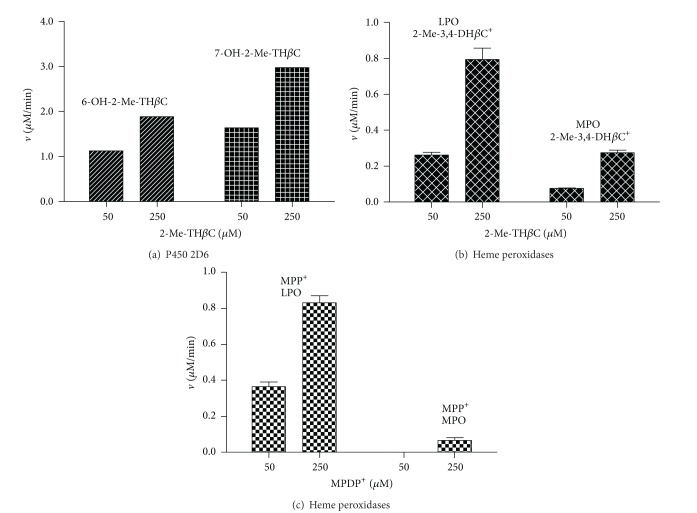
Metabolites and rates produced from 2-methyl-1,2,3,4-tetrahydro-*β*-carboline (2-Me-TH*β*C) by cytochrome P450 2D6 (a) and lactoperoxidase (LPO) and myeloperoxidase (MPO) (b). (c) Corresponds to the formation of MPP^+^ from MPDP^+^ as catalyzed by peroxidases LPO and MPO (the corresponding controls with H_2_O_2_ and no enzyme are subtracted). Enzyme assays were as indicated in experimental section.

 In a search for analogies with MPTP, the naturally occurring *β*-carboline 2-methyltetrahydro-*β*-carboline was metabolized by the former enzymes (Figure 2). Human MAO enzymes (MAO-A or -B) did not afford any detectable metabolites of oxidation (i.e., *β*-carbolinium species). Therefore, MPTP and its *β*-carboline analogs behaved differently regarding the metabolism by MAO, suggesting that they differ in the activation pathway (Figure 2). Instead, the cytochrome P450 2D6 was able to metabolize the tetrahydro-*β*-carboline that was hydroxylated to two metabolites identified as 6-hydroxy-2-methyl-1,2,3,4-tetrahydro-*β*-carboline (6-OH-2-Me-TH*β*C) and 7-hydroxy-2-methyl-1,2,3,4-tetrahydro-*β*-carboline (7-OH-2-Me-TH*β*C) (Figures 5(a) and 6(a)). These polar metabolites could be considered detoxification metabolites, and, in that case, the cytochrome P450 2D6 can participate in an inactivation route of 2-methyltetrahydro-*β*-carboline, in a similar way to MPTP. On the other hand, 2-methyltetrahydro-*β*-carboline was oxidized in a reaction catalyzed by heme peroxidases in the presence of H_2_O_2_ (Figure 5(b)). This tetrahydro-*β*-carboline was oxidized by lactoperoxidase and myeloperoxidase to the corresponding 2-methyl-3,4-dihydro-*β*-carbolinium cation (2-Me-3,4-DH*β*C^+^) (Figure 6(b)) and traces detected of the fully aromatic *β*-carbolinium cation (2-methyl-*β*-carbolinium cation). The *β*-carbolinium species are neurotoxic substances [30, 33], and therefore this oxidation may represent a new route of activation of naturally occurring 2-methyltetrahydro-*β*-carbolines, which could be of significance for the toxicological fate of these substances (Figure 2). These results agree with the ability of tetrahydro-*β*-carbolines to be oxidized to dihydro- and aromatic *β*-carbolines [27, 59] and also with the participation of these substrates in a reduction of redox intermediates of peroxidases [53]. 

MPTP induces parkinsonism in humans and animal models, whereas the *β*-carbolines were postulated as possible toxins involved in neurodegeneration [20]. As seen in Figure 2, biochemical reactions leading to the activation/inactivation of these substances are critical for their toxicological outcome. A so-called “activation” to toxic pyridinium or *β*-carbolinium species is required for toxicity, whereas an “inactivation” may influence the fate of these protoxins in the body. Differences in the activation/inactivation balance and consequently in the response to these substances may arise from differences in the enzymes involved. The toxic response to MPTP largely varies between species [7, 10, 11], and this might result from differences in the expression and activity of metabolic enzymes [49, 50, 60, 61] producing a different ratio between toxic and inactive metabolites. The results reported here indicate substantial differences among the enzymes involved and profile (Figure 2). While MAO enzymes were responsible for the activation of MPTP to give toxic pyridinium species, heme peroxidases were activators of 2-methyltetrahydro-*β*-carbolines to pyridinium-like carbolinium toxins and MAO played no role. Although heme peroxidases were not involved in the MPTP activation, these enzymes catalyzed the oxidation step from MPDP^+^ to MPP^+^. Thus, heme peroxidases like myeloperoxidase may accelerate the flow from MPDP^+^ (i.e., produced by MAO) to MPP^+^. With these results in mind, peroxidases might play a role in the bioactivation of these or related protoxins, resulting in increased toxicity (Figure 2) [15]. In this regard, the potential involvement of peroxidases in neurodegeneration and Parkinson's disease has been already suggested [50–52, 62]. Myeloperoxidase occurs at sites of degenerative diseases and neuroinflammation and increases in Alzheimer's disease [48, 63], and its ablation mitigated PD produced by MPTP neurotoxin in animals [49]. 

MPTP and 2-methyltetrahydro-*β*-carbolines differed in the activation route to toxic metabolites (i.e., MAO versus heme peroxidase). However, both were metabolized by human cytochrome P450 2D6. This enzyme carried out the metabolism of MPTP by *N*-demethylation and aromatic hydroxylation and 2-methyltetrahydro-*β*-carboline by aromatic hydroxylation (Figure 2). The involvement of cytochrome P450 2D6 in detoxification is relevant, and some studies have reported an association between cytochrome P450 2D6 polymorphism and Parkinson's disease (PD) [44, 64]. This enzyme is lower in PD patients, which may reduce the ability of those patients to inactivate PD-causing neurotoxicity [64]. For example, exposure to pesticides increases the incidence of PD, and this risk was even higher in subjects with a poor metabolizer 2D6 genotype exposed to pesticides [3, 45]. In contrast to pyridinium species (i.e., MPDP^+^ and MPP^+^) produced by MAO, the MPTP-OH and PTP metabolites arising from cytochrome P450 2D6 are thought to be devoid of neurotoxicity [12, 65]. Therefore, cytochrome P450 2D6 competes with MAO enzymes in favour of an inactivation route of the MPTP neurotoxin [14]. Results in Figure 4 also showed that P450 2D6 slightly activated MPTP to the pyridinium species, MPDP^+^ and MPP^+^. Recently, a mitochondrial cytochrome P450 2D6 was reported that was able to carry out the activation of MPTP to pyridinium species (MPDP^+^ and MPP^+^) suggesting a role for this enzyme in the activation process and toxicity [17]; however, this conversion appeared to be of lower significance compared with MAO [14]. 

*β*-Carbolines abound in plants and foods and appear in biological tissues including the brain [25, 27, 66, 67]. They exert psychopharmacological and behavioural effects [23–27, 54, 66, 68]. An involvement of tetrahydro-*β*-carbolines (and/or *β*-carbolines) as proneurotoxins is based on the fact that these compounds are *N*-methylated to *N*(2)-methyltetrahydro-*β*-carbolines which can be subsequently oxidized to *N*(2)-methyl-*β*-carbolinium species [20, 21, 29, 33, 69]. *N*-Methylation of tetrahydro-*β*-carbolines (TH*β*C) is catalyzed by brain *N*-methyltransferases [69], and 2-methyltetrahydro-*β*-carboline has been detected in rat brain [70]. However, this sequence lacks a step of activation to *β*-carbolinium species. 2-Methyltetrahydro-*β*-carboline is not neurotoxic [65] but it could be oxidized (activated) to toxic *β*-carbolinium cation (*β*C^+^) by heme peroxidases/H_2_O_2_ (lactoperoxidase and myeloperoxidase) (Figures 2 and 6). *β*-Carbolinium species resemble MPP^+^ in many of their toxicological features. *N*-Methyl-*β*-carbolinium cation (2-Me-*β*C^+^) and *N*,*N*-dimethyl-*β*-carbolinium cation (2,9-diMe-*β*C^+^) are neurotoxins producing irreversible striatal lesions [31, 71]. These carbolinium species (2-Me-*β*C^+^ and 2,9-diMe-*β*C^+^) were found in normal and parkinsonian brains [34–36] and appeared in higher proportion in the brain and cerebrospinal fluid of PD patients [34, 35]. As the cytochrome P450 2D6 contributes to the metabolism of tetrahydro-*β*-carbolines to hydroxylated metabolites [14, 43], this route could be competitive against the oxidation by peroxidases and the *N*-methylation by *N*-methyltransferases [14, 15, 21]. The biotransformation of *β*-carbolines by cytochrome P450 2D6 may influence the outcome of these substances as eventual protoxins and generate differences depending on enzyme polymorphism.

In summary, these results indicate that activation/inactivation of MPTP and 2-methyltetrahydro-*β*-carboline protoxins depends on three key oxidative enzymes that are crucial for toxicity and detoxification. MPTP relays on MAO enzymes for activation (toxicity) with a possible role for heme peroxidases. However, it relays on cytochrome P450 2D6 for inactivation. In contrast, 2-methyltetrahydro-*β*-carbolines depend on heme peroxidases for activation to toxic carbolinium species and cytochrome P450 2D6 for detoxification with no role played by MAO enzymes. It can be concluded that the degree of toxicity caused by those protoxins may result from a balance between the rate of activation to toxic products (i.e., *N*-methylpyridinium—MPP^+^ and MPDP^+^—and *N*-methyl-*β*-carbolinium—*β*C^+^—) and the rate of inactivation (detoxification) (i.e., *N*-demethylation, aromatic hydroxylation). As the enzymes involvement may vary in expression and activity between persons, major differences in the toxicological outcome of these protoxins are foreseen.

## References

[1] Langston JW, Ballard P, Tetrud JW, Irwin I (1983). Chronic parkinsonism in humans due to a product of meperidine-analog synthesis. *Science*.

[2] Cannon JR, Greenamyre JT (2011). The role of environmental exposures in neurodegeneration and neurodegenerative diseases. *Toxicological Sciences*.

[3] Moretto A, Colosio C (2011). Biochemical and toxicological evidence of neurological effects of pesticides: the example of Parkinson’s disease. *NeuroToxicology*.

[4] Jackson-Lewis V, Przedborski S (2007). Protocol for the MPTP mouse model of Parkinson’s disease. *Nature Protocols*.

[5] Kidd SK, Schneider JS (2011). Protective effects of valproic acid on the nigrostriatal dopamine system in a 1-methyl-4-phenyl-1,2,3,6-tetrahydropyridine mouse model of Parkinson’s disease. *Neuroscience*.

[6] Mythri RB, Veena J, Harish G, Rao BSS, Bharath MMS (2011). Chronic dietary supplementation with turmeric protects against 1-methyl-4-phenyl-1,2,3,6-tetrahydropyridine-mediated neurotoxicity in vivo: implications for Parkinson’s disease. *British Journal of Nutrition*.

[7] Blandini F, Armentero M-T (2012). Animal models of Parkinson’s disease. *FEBS Journal*.

[8] Langston JW, Irwin I, Langston EB, Forno LS (1984). 1-methyl-4-phenylpyridinium ion (MPP^+^): identification of a metabolite of MPTP, a toxin selective to the substantia nigra. *Neuroscience Letters*.

[9] Lehner A, Johnson M, Simkins T (2011). Liquid chromatographic-electrospray mass spectrometric determination of 1-methyl-4-phenylpyridine (MPP^+^) in discrete regions of murine brain. *Toxicology Mechanisms and Methods*.

[10] Ito T, Uchida K, Nakayama H (2013). Neuronal or inducible nitric oxide synthase (NOS) expression level is not involved in the different susceptibility to nigro-striatal dopaminergic neurotoxicity induced by 1-methyl-4-phenyl-1,2,3,6-tetrahydropyridine (MPTP) between C57BL/6 and BALB/c mice. *Experimental and Toxicologic Pathology*.

[11] Ito T, Suzuki K, Uchida K, Nakayama H (2012). 1-methyl-4-phenyl-1,2,3,6-tetrahydropyridine (MPTP)-induced neuroblastic apoptosis in the subventricular zone is caused by 1-methyl-4-phenylpyridinium (MPP^+^) converted from MPTP through MAO-B. *Experimental and Toxicologic Pathology*.

[12] Gilham DE, Cairns W, Paine MJI (1997). Metabolism of MPTP by cytochrome P4502D6 and the demonstration of 2D6 mRNA in human foetal and adult brain by in situ hybridization. *Xenobiotica*.

[13] Weissman J, Trevor A, Chiba K (1985). Metabolism of the nigrostriatal toxin 1-methyl-4-phenyl-1,2,3,6-tetrahydropyridine by liver homogenate fractions. *Journal of Medicinal Chemistry*.

[14] Herraiz T, Guillén H, Arán VJ, Idle JR, Gonzalez FJ (2006). Comparative aromatic hydroxylation and *N*-demethylation of MPTP neurotoxin and its analogs, *N*-methylated *β*-carboline and isoquinoline alkaloids, by human cytochrome P450 2D6. *Toxicology and Applied Pharmacology*.

[15] Herraiz T, Guillén H, Galisteo J (2007). *N*-methyltetrahydro-*β*-carboline analogs of 1-methyl-4-phenyl-1,2,3,6-tetrahydropyridine (MPTP) neurotoxin are oxidized to neurotoxic *β*-carbolinium cations by heme peroxidases. *Biochemical and Biophysical Research Communications*.

[16] Modi S, Gilham DE, Sutcliffe MJ (1997). 1-methyl-4-phenyl-1,2,3,6-tetrahydropyridine as a substrate of cytochrome P450 2D6: allosteric effects of NADPH-cytochrome P450 reductase. *Biochemistry*.

[17] Bajpai P, Sangar MC, Singh S (2013). Metabolism of 1-methyl-4-phenyl-1,2,3,6-tetrahydropyridine by mitochondrion-targeted cytochrome P450 2D6: implications in Parkinson disease. *The Journal of Biological Chemistry*.

[18] Hanna IH, Krauser JA, Cai H, Kim M-S, Guengerich FP (2001). Diversity in mechanisms of substrate oxidation by cytochrome P450 2D6: lack of an allosteric role of NADPH-cytochrome P450 reductase in catalytic regioselectivity. *The Journal of Biological Chemistry*.

[19] Coleman T, Ellis SW, Martin IJ, Lennard MS, Tucker GT (1996). 1-methyl-4-phenyl-1,2,3,6-tetrahydropyridine (MPTP) is *N*-demethylated by cytochromes P450 2D6, 1A2 and 3A4—implications for susceptibility to Parkinson’s disease. *Journal of Pharmacology and Experimental Therapeutics*.

[20] Collins MA, Neafsey EJ (1985). *β*-carboline analogues of *N*-methyl-4-phenyl-1,2,5,6-tetrahydropyridine (MPTP): endogenous factors underlying idiopathic parkinsonism?. *Neuroscience Letters*.

[21] Herraiz T, Antkiewicz-Michaluk L, Rommelspacher H (2012). *β*-carbolines as neurotoxins. *Isoquinolines and *β*-Carbolines as Neurotoxins and Neuroprotectants: New Vistas in Parkinson’s Disease Therapy*.

[22] Herraiz T (2004). Relative exposure to *β*-carbolines norharman and harman from foods and tobacco smoke. *Food Additives and Contaminants*.

[23] Robinson ESJ, Anderson NJ, Crosby J, Nutt DJ, Hudson AL (2003). Endogenous *β*-carbolines as clonidine-displacing substances. *Annals of the New York Academy of Sciences*.

[24] Herraiz T, Chaparro C (2005). Human monoamine oxidase is inhibited by tobacco smoke: *β*-carboline alkaloids act as potent and reversible inhibitors. *Biochemical and Biophysical Research Communications*.

[25] Herraiz T, Chaparro C (2006). Human monoamine oxidase enzyme inhibition by coffee and *β*-carbolines norharman and harman isolated from coffee. *Life Sciences*.

[26] Airaksinen MM, Kari I (1981). *β*-carbolines, psychoactive compounds in the mammalian body—part I: occurrence, origin and metabolism. *Medical Biology*.

[27] Herraiz T, Galisteo J (2003). Tetrahydro-*β*-carboline alkaloids occur in fruits and fruit juices. Activity as antioxidants and radical scavengers. *Journal of Agricultural and Food Chemistry*.

[28] Herraiz T (1998). Occurrence of 1,2,3,4-tetrahydro-*β*-carboline-3-carboxylic acid and 1-methyl-1,2,3,4-tetrahydro-*β*-carboline-3-carboxylic acid in fruit juices, purees, and jams. *Journal of Agricultural and Food Chemistry*.

[29] Gearhart DA, Collins MA, Lee JM, Neafsey EJ (2000). Increased *β*-carboline 9*N*-methyltransferase activity in the frontal cortex in Parkinson’s disease. *Neurobiology of Disease*.

[30] Wernicke C, Schott Y, Enzensperger C, Schulze G, Lehmann J, Rommelspacher H (2007). Cytotoxicity of *β*-carbolines in dopamine transporter expressing cells: structure-activity relationships. *Biochemical Pharmacology*.

[31] Pavlovic S, Schulze G, Wernicke C (2006). 2,9-dimethyl-*β*-carbolinium, a neurotoxin occurring in human brain, is a potent inducer of apoptosis as 1-methyl-4-phenylpyridinium. *Neuroscience*.

[32] Lorenc-Koci E, Rommelspacher H, Schulze G (2006). Parkinson’s disease-like syndrome in rats induced by 2,9-dimethyl-*β*- carbolinium ion, a *β*-carboline occurring in the human brain. *Behavioural Pharmacology*.

[33] Storch A, Hwang Y-I, Gearhart DA (2004). Dopamine transporter-mediated cytotoxicity of *β*-carbolinium derivatives related to Parkinson’s disease: relationship to transporter-dependent uptake. *Journal of Neurochemistry*.

[34] Matsubara K, Collins MA, Akane A (1993). Potential bioactivated neurotoxicants, *N*-methylated *β*-carbolinium ions, are present in human brain. *Brain Research*.

[35] Matsubara K, Kobayashi S, Kobayashi Y (1995). *β*-carbolinium cations, endogenous MPP^+^ analogs, in the lumbar cerebrospinal fluid of patients with Parkinson’s disease. *Neurology*.

[36] Matsubara K, Gonda T, Sawada H (1998). Endogenously occurring *β*-carboline induces parkinsonism in nonprimate animals: a possible causative protoxin in idiopathic Parkinson’s disease. *Journal of Neurochemistry*.

[37] Youdim MBH, Edmondson D, Tipton KF (2006). The therapeutic potential of monoamine oxidase inhibitors. *Nature Reviews Neuroscience*.

[38] Naoi M, Maruyama W, Inaba-Hasegawa K (2012). Type A and B monoamine oxidase in age-related neurodegenerative disorders: their distinct roles in neuronal death and survival. *Current Topics in Medicinal Chemistry*.

[39] Lieu CA, Chinta SJ, Rane A, Andersen JK (2013). Age-related behavioral phenotype of an astrocytic monoamine oxidase-B transgenic mouse model of Parkinson's Disease. *PLoS ONE*.

[40] Weinreb O, Amit T, Bar-Am O, Youdim MBH (2010). Rasagiline: a novel anti-Parkinsonian monoamine oxidase-B inhibitor with neuroprotective activity. *Progress in Neurobiology*.

[41] Mallajosyula JK, Kaur D, Chinta SJ (2008). MAO-B elevation in mouse brain astrocytes results in Parkinson’s pathology. *PLoS ONE*.

[42] Yu A-M, Idle JR, Byrd LG, Krausz KW, Küpfer A, Gonzalez FJ (2003). Regeneration of serotonin from 5-methoxytryptamine by polymorphic human CYP2D6. *Pharmacogenetics*.

[43] Yu A-M, Idle JR, Herraiz T, Küpfer A, Gonzalez FJ (2003). Screening for endogenous substrates reveals that CYP2D6 is a 5-methoxyindolethylamine *O*-demethylase. *Pharmacogenetics*.

[44] McCann SJ, Pond SM, James KM, le Couteur DG (1997). The association between polymorphisms in the cytochrome P-450 2D6 gene and Parkinson’s disease: a case-control study and meta-analysis. *Journal of the Neurological Sciences*.

[45] Elbaz A, Levecque C, Clavel J (2004). CYP2D6 polymorphism, pesticide exposure, and Parkinson’s disease. *Annals of Neurology*.

[46] Mann A, Tyndale RF (2010). Cytochrome P450 2D6 enzyme neuroprotects against 1-methyl-4-phenylpyridinium toxicity in SH-SY5Y neuronal cells. *European Journal of Neuroscience*.

[47] Tafazoli S, O’Brien PJ (2005). Peroxidases: a role in the metabolism and side effects of drugs. *Drug Discovery Today*.

[48] Green PS, Mendez AJ, Jacob JS (2004). Neuronal expression of myeloperoxidase is increased in Alzheimer’s disease. *Journal of Neurochemistry*.

[49] Choi D-K, Pennathur S, Perier C (2005). Ablation of the inflammatory enzyme myeloperoxidase mitigates features of Parkinson’s disease in mice. *Journal of Neuroscience*.

[50] Huh SH, Chung YC, Piao Y (2011). Ethyl pyruvate rescues nigrostriatal dopaminergic neurons by regulating glial activation in a mouse model of Parkinson’s disease. *Journal of Immunology*.

[51] Galzigna L, Schiappelli MP, Rigo A, Scarpa M (1999). A rat brain fraction and different purified peroxidases catalyzing the formation of dopaminochrome from dopamine. *Biochimica et Biophysica Acta*.

[52] Everse J, Coates PW (2005). Role of peroxidases in Parkinson disease: a hypothesis. *Free Radical Biology and Medicine*.

[53] Jantschko W, Furtmüller PG, Allegra M (2002). Redox intermediates of plant and mammalian peroxidases: a comparative transient-kinetic study of their reactivity toward indole derivatives. *Archives of Biochemistry and Biophysics*.

[54] Herraiz T, Guillén H (2011). Inhibition of the bioactivation of the neurotoxin MPTP by antioxidants, redox agents and monoamine oxidase inhibitors. *Food and Chemical Toxicology*.

[55] Heikkila RE, Manzino L, Cabbat FS, Duvoisin RC (1984). Protection against the dopaminergic neurotoxicity of 1-methyl-4-phenyl-1,2,5,6-tetrahydropyridine by monoamine oxidase inhibitors. *Nature*.

[56] Herraiz T (2012). Evaluation of the oxidation of 1-methyl-4-phenyl-1,2,3,6-tetrahydropyridine (MPTP) to toxic pyridinium cations by monoamine oxidase (MAO) enzymes and its use to search for new MAO inhibitors and protective agents. *Journal of Enzyme Inhibition and Medicinal Chemistry*.

[57] di Monte DA, Wu EY, Irwin I, Delanney LE, Langston JW (1991). Biotransformation of 1-methyl-4-phenyl-1,2,3,6-tetrahydropyridine in primary cultures of mouse astrocytes. *Journal of Pharmacology and Experimental Therapeutics*.

[58] Peterson LA, Caldera PS, Trevor A, Chiba K, Castagnoli N (1985). Studies on the 1-methyl-4-phenyl-2,3-dihydropyridinium species 2,3-MPDP^+^, the monoamine oxidase catalyzed oxidation product of the nigrostriatal toxin 1-methyl-4-phenyl-1,2,3,6-tetrahydropyridine (MPTP). *Journal of Medicinal Chemistry*.

[59] Herraiz T, Galisteo J (2002). Tetrahydro-*β*-carboline alkaloids that occur in foods and biological systems act as radical scavengers and antioxidants in the ABTS assay. *Free Radical Research*.

[60] Jimenez-Jimenez FJ, Tabernero C, Mena MA (1991). Acute effects of 1-methyl-4-phenyl-1,2,3,6-tetrahydropyridine in a model of rat designated a poor metabolizer of debrisoquine. *Journal of Neurochemistry*.

[61] Inoue H, Castagnoli K, van der Schyf C, Mabic S, Igarashi K, Castagnoli N (1999). Species-dependent differences in monoamine oxidase A and B-catalyzed oxidation of various C4 substituted 1-methyl-4-phenyl-1,2,3,6- tetrahydropyridinyl derivatives. *Journal of Pharmacology and Experimental Therapeutics*.

[62] Everse J, Liu C-JJ, Coates PW (2011). Physical and catalytic properties of a peroxidase derived from cytochrome c. *Biochimica et Biophysica Acta*.

[63] Lefkowitz DL, Lefkowitz SS (2008). Microglia and myeloperoxidase: a deadly partnership in neurodegenerative disease. *Free Radical Biology and Medicine*.

[64] Mann A, Miksys SL, Gaedigk A, Kish SJ, Mash DC, Tyndale RF (2012). The neuroprotective enzyme CYP2D6 increases in the brain with age and is lower in Parkinson’s disease patients. *Neurobiology of Aging*.

[65] Perry TL, Jones K, Hansen S, Wall RA (1987). 4-phenylpyridine and three other analogues of 1-methyl-4-phenyl-1,2,3,6-tetrahydropyridine lack dopaminergic nigrostriatal neurotoxicity in mice and marmosets. *Neuroscience Letters*.

[66] Herraiz T, González D, Ancín-Azpilicueta C, Arán VJ, Guillén H (2010). *β*-carboline alkaloids in *Peganum harmala* and inhibition of human monoamine oxidase (MAO). *Food and Chemical Toxicology*.

[67] Herraiz T (2000). Tetrahydro-*β*-carboline-3-carboxylic acid compounds in fish and meat: possible precursors of co-mutagenic *β*-carbolines norharman and harman in cooked fools. *Food Additives and Contaminants*.

[68] Louis ED, Rios E, Pellegrino KM, Jiang W, Factor-Litvak P, Zheng W (2008). Higher blood harmane (1-methyl-9H-pyrido[3,4-b]indole) concentrations correlate with lower olfactory scores in essential tremor. *NeuroToxicology*.

[69] Matsubara K, Collins MA, Neafsey EJ (1992). Mono-*N*-methylation of 1,2,3,4-tetrahydro-*β*-carbolines in brain cytosol: absence of indole methylation. *Journal of Neurochemistry*.

[70] Barker SA, Harrison REW, Monti JA, Brown GB, Christian ST (1981). Identification and quantification of 1,2,3,4-tetrahydro-*β*-carboline, 2-methyl-1,2,3,4-tetrahydro-*β*-carboline and 6-methoxy-1,2,3,4-tetrahydro-*β*-carboline as *in vivo* constituents of rat brain and adrenal gland. *Biochemical Pharmacology*.

[71] Hamann J, Rommelspacher H, Storch A, Reichmann H, Gille G (2006). Neurotoxic mechanisms of 2,9-dimethyl-*β*-carbolinium ion in primary dopaminergic culture. *Journal of Neurochemistry*.

